# Correlation of fibrinogen-like protein 2 with disease progression in patients with severe acute pancreatitis

**DOI:** 10.3892/etm.2013.1354

**Published:** 2013-10-22

**Authors:** XIAOHUA YE, JIAPING HUAI, RENPIN CHEN, JIN DING, YANPING CHEN, ZHENZHAI CAI

**Affiliations:** 1 Department of Gastroenterology and Hepatology, Jinhua Municipal Central Hospital, Jinhua Hospital of Zhejiang University, Jinhua, Zhejiang 321000, P.R. China; 2 Department of Critical Care Medicine, Jinhua Municipal Central Hospital, Jinhua Hospital of Zhejiang University, Jinhua, Zhejiang 321000, P.R. China; 3 Department of Gastroenterology and Hepatology, the First Affiliated Hospital of Wenzhou Medical University, Wenzhou, Zhejiang 325000, P.R. China; 4 Department of Gastroenterology, the Second Affiliated Hospital of Wenzhou Medical University, Wenzhou, Zhejiang 325000, P.R. China

**Keywords:** fibrinogen-like protein 2, severe acute pancreatitis, ranson, acute physiology and chronic health evaluation II

## Abstract

It has recently been demonstrated that fibrinogen-like protein 2 (fgl2) is expressed on the surface of macrophages, T cells and endothelial cells and directly cleaves prothrombin to thrombin. The present study was designed to examine fgl2 expression in patients with severe acute pancreatitis (SAP) and its correlation with disease progression. Peripheral blood mononuclear cells (PBMCs) were isolated from 25 patients with SAP, 37 patients with mild acute pancreatitis (MAP) and 20 healthy volunteers as controls. Paraffin sections of pancreas were obtained from 18 postoperative patients with SAP between 2003 and 2012. Human fgl2 (hfgl2) gene expression was determined in the PBMCs by real-time PCR. A monoclonal antibody against hfgl2 was applied to detect hfgl2 protein expression in the pancreatic tissues as well as in the PBMCs by immunohistochemical staining. The levels of hfgl2 expression in the PBMCs from the 25 patients with SAP were markedly upregulated compared with the other groups, whereas no significant difference between the MAP group and healthy controls was observed. hfgl2 expression in the PBMCs and pancreatic tissues was detectable through using immunohistochemistry and was demonstrated to be specifically localized to the endothelium of microvessels and inflammatory infiltrative cells in the areas of acute focal, confluent necrosis. There were positive correlations between hfgl2 expression in the PBMCs and the severity of SAP, as indicated by scores of Ranson and Acute Physiology and Chronic Health Evaluation II. The results suggest that hfgl2 is involved in the pathogenesis of SAP and hfgl2 levels may serve as a biomarker during disease progression.

## Introduction

The hallmark of severe acute pancreatitis (SAP) is extreme rapidity of disease progression with a high risk of mortality, ranging from 30 to 50% (1). The aetiology of SAP is heterogeneous and involves a complex cascade of events, however, the exact pathophysiological mechanisms remain a subject of debate.

It is believed that the coagulation dysregulation and thromboembolism that occur during disease progression are critical in the pathogenesis of SAP, which is associated with its severity (2,3). The hemostatic system is activated in the course of SAP with the production of microthrombi in the microvessels, and pathological changes ranging from scattered intravascular thrombosis to disseminated intravascular coagulation are integral to coagulative disorders (4). Severe complications, such as multiple organ dysfunction syndrome, which are due to microcirculatory disturbances and microvascular thrombosis, originate from vascular endothelial cell injuries and hypercoagulation (5). Adding to the complexity, there are interactions between acute inflammatory mediators, which are released from the inflammation cascade during SAP and the coagulation system, suggesting that the two events are closely linked processes (1–3,6). In turn, the inflammation-mediated initiation of coagulation (1,2,6), fibrin deposition and formation of thrombin contribute to the local inflammatory response (7). Treatment with systemic anti-coagulants may improve the microcirculatory perfusion and are capable of exerting an anti-inflammatory effect (7,8). However, the precise mechanisms remain to be determined.

Fibrinogen-like protein 2 (fgl2), also known as fgl2 prothrombinase, is a novel member of the fibrinogen-associated protein superfamily, which includes tenascin and angiopoietin (9). Fgl2 is expressed and differentially regulated in various cell types (10). Fgl2 has been suggested as crucial in microthrombosis and its biological characteristics, in line with coagulation factor Xa, may cleave prothrombinase into activated thrombinase, thereby initiating microthrombosis (11,12). Fgl2 is involved in viral hepatitis (11,13), acute vascular xenograft rejection (14,15) and cytokine-induced fetal loss syndrome (16,17) by mediating ‘immune coagulation’ (11), which facilitates microthrombosis, resulting in microvascular disturbance. However, whether human fgl2 (hfgl2) is involved in patients with SAP remains to be elucidated.

In the present study, the expression and histological localization of hfgl2 were detected in peripheral blood mononuclear cells (PMBCs) and pancreatic tissues and its correlation with disease progression was assessed in patients with SAP, with the expectation of providing a novel point of view on the pathogenesis of SAP and a novel biomarker for predicting the occurrence of SAP.

## Materials and methods

### Patients and collection of specimens

The definition of SAP is based on the typical clinical features, laboratory evidence, typical manifestation during ultrasonography and contrast-enhanced computed tomography (CT), and intraoperative findings. All participants signed consent forms for this study and the study protocol was reviewed and approved by the Institutional Review Board of the First Affiliated Hospital of Wenzhou Medical College (Wenzhou, China). Samples from 25 patients diagnosed with SAP (11 male and 14 female; mean age 62.6 years, range 21–83 years) and 37 patients diagnosed with mild acute pancreatitis (MAP) (17 male and 20 female; mean age 59.0 years, range 19–85 years), who were admitted to the hospital within 24–48 h of the onset of disease, were collected from June 2011 to September 2012. The etiology of the cases of MAP and SAP are presented in Table I. In addition, 20 healthy volunteers (9 male and 11 female; mean age 58.95 years, range 23–84 years) were recruited as healthy controls. No significant differences in age and gender among the three groups were detected (Table II).

All clinical definitions complied with the Atlanta classification system for acute pancreatitis (18). Evaluations of scores of Ranson and APACHE II were performed within 24 h after admission. PBMCs were freshly isolated from the 82 participants and stored at −80ºC for subsequent use. Paraffin sections were also reviewed and obtained for pancreatic tissues from 10 postoperative patients (7 male and 3 female; mean age 53.2 years, range 34–77 years) with SAP and eight (3 male and 5 female; mean age 50.8 years, range 35–71 years) with MAP between 2003 and 2011, respectively. Of the eight MAP cases, three were patients with pancreatic cancers, as the normal tissues other than the tumor tissues were collected, and the other five cases were patients with gallstones.

### Real-time quantitative PCR (qPCR)

Total RNA was isolated from the PBMCs using TRIzol reagent (Invitrogen, Carlsbad, CA, USA) according to the manufacturer’s instructions. The first-strand cDNA was subsequently synthesized (MBI Fermentas, Burlington, Canada). The PCR primer sequences (Generay Biotech Co., Ltd., Shanghai, China) used were as follows: 5-CCTGGAGATTGTGGTTTCGT-3 and 5-TACCATGCCTTTCTCCAAGG-3 for hfgl2; and 5-TGTCACCAACTGGGACGATA-3 and 5-GGGGTGTTGAAGGTCTCAAA-3 for β-actin, which was used as an internal control. The qPCR was performed with the ABI 7500 Sequence Detection system (Applied Biosystems, Carlsbad, CA, USA) according to the manufacturer’s instructions. The cDNA was amplified over 40 cycles, denatured at 95ºC for 15 sec, annealed at 60ºC for 45 sec and extended at 72ºC for 60 sec. The samples were run in triplicate and the relative expression was detected by normalizing to the β-actin levels. The expression levels of the targeted genes were calculated by using the 2^−ΔΔCT^ method.

### Immunohistochemical staining of hfgl2

Immunohistochemical staining was performed to evaluate the expression of hfgl2 in the pancreatic tissues and PBMCs. Paraffin-embedded pancreatic tissues (4 μm) were sectioned. Microwave antigen retrieval was conducted for 20 min in citrate buffer (pH 6.0) to activate antigens. Blocking of endogenous peroxidase was achieved using 0.3% H_2_O_2_ at room temperature for 10 min. The pancreatic tissue slices or PBMCs were incubated with mouse anti-fgl2 monoclonal antibody (Abnova Corp., Taipei, Taiwan) at 4ºC overnight. Subsequently, the slices were incubated with a secondary antibody (Zhongshan Goldenbridge, Beijing, China) at 37ºC for 30 min, followed by developing with diaminobenzidine and counterstaining with hematoxylin. PBS was used instead of the primary antibody as the negative control.

### Statistical analysis

Data were expressed as the mean ± standard deviation. Statistical Program for the Social Sciences Software, version 15.0 (SPSS, Inc., Chicago, IL, USA) was used to conduct the analysis. Statistical analysis was performed by one-way analysis of variance. The Spearman grade correlation analysis was used to evaluate the associations among the parameters. P≤0.05 was considered to indicate a statistically significant difference.

## Results

### Upregulated hfgl2 expression in PBMCs from patients with SAP

**Figure 1:**
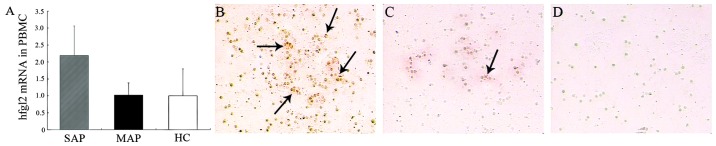
Levels of hfgl2 mRNA and protein expression in the PBMCs. (A) The calculated levels of hfgl2 mRNA in the PBMCs. The expression of hfgl2 mRNA is the relative ratio to β-actin. (B–D) Hfgl2 protein expression in the PBMCs of the patients with (B) SAP and (C) MAP and the (D) HCs. Arrows indicate hfgl2-positive cells. Data are expressed as mean ± SD. Hfgl2, human fibrinogen-like protein 2; PBMC, peripheral blood mononuclear cell; SAP, severe acute pancreatitis; MAP, mild acute pancreatitis; HC, healthy control.

Hfgl2 expression in the PBMCs was assessed by qPCR analysis and immunohistochemistry. The levels of hfgl2 mRNA were significantly (P<0.01) upregulated in the patients with SAP compared with those in the patients with MAP and the healthy controls, whereas there was no significant difference (P>0.05) between those of the MAP group and the healthy controls (Fig. 1A). Hfgl2 protein was highly detectable in the PBMCs from the patients with SAP, in those from the MAP group or the healthy controls (Fig. 1B–D).

### Hfgl2 expression in pancreatic tissues

**Figure 2:**
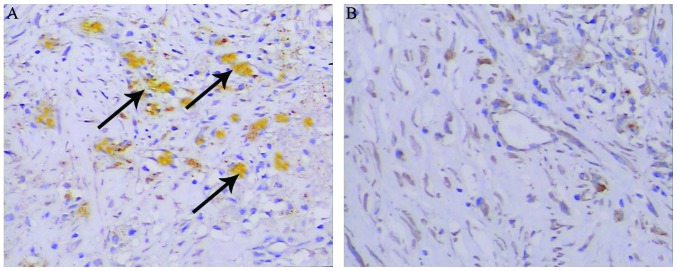
Hfgl2 expression in the pancreatic tissues of patients with (A) SAP and (B) MAP, identified by immunochemical staining (×200). Arrows indicate hfgl2-positive cells. Hfgl2, human fibrinogen-like protein 2; SAP, severe acute pancreatitis; MAP, mild acute pancreatitis.

Paraffin sections were reviewed and collected from postoperative patients with SAP or MAP to evaluate hfgl2 expression in pancreatic tissues. Results of immunohistochemistry demonstrated that hfgl2 was primarily localized in infiltrating interstitial and endothelial cells of the microvasculature, whereas little fgl2 was identified in the pancreatic tissues of the patients with MAP (Fig. 2).

### Hfgl2 expression correlates with the severity of SAP

**Figure 3:**
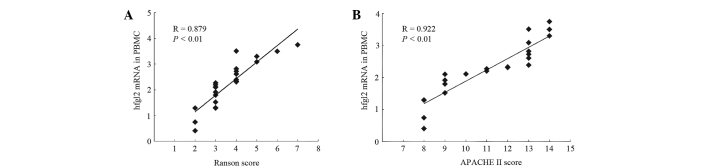
Correlation of hfgl2 mRNA levels in the PBMCs with severity indexes calculated on admission. Correlation with (A) Ranson and (B) APACHE II scores. Hfgl2, human fibrinogen-like protein 2; PBMC, peripheral blood mononuclear cell; APACHE, Acute Physiology and Chronic Health Evaluation.

The Ranson and APACHE II scores were significantly elevated in the SAP group compared with the MAP group (Table III). Spearman grade correlation analysis was conduced to determine the strength of the association between fgl2 expression and the severity of SAP, as indicated by the scoring systems. The data demonstrated that there was a strong correlation between fgl2 expression and the scores of Ranson (r=0.937, P<0.01) and APACHE II (r=0.976, P<0.01) (Fig. 3).

## Discussion

SAP is characterized as an inflammatory disease, in which dysregulated cytokines such as TNF-α may ‘drive’ the progression of this disorder (2,3). During this process, the inflammation and coagulation cascade are associated (1,3–6). Furthermore, microthrombosis due to fibrin deposition has been demonstrated to be pivotal in the pathogenesis of SAP and occurs early on the onset (2,3,19). In the present study, an important role for hfgl2 was defined in the generation of fibrin and subsequently the formation of microthrombi in patients with SAP.

Procoagulants other than tissue factor, which are induced specifically by immune mediators, may be critical in promoting localized fibrin deposition and result in disturbance of microcirculation (20). Fgl2/fibroleukin, a novel procoagulant, is a 70-kDa type-2 transmembrane protein containing a C-terminal fibrinogen-related extracellular domain which has been found to directly cleave prothrombin to thrombin in an alternative way and be crucial in microthrombosis (9,11,12). Moreover, fgl2 was demonstrated to be primarily expressed on activated macrophages, T cells and endothelial cells (21). In the present study, marked hfgl2 expression as a source of procoagulant activity was identified in PBMCs (containing lymphocytes, monocytes, dendritic cells and a few other types of cell) from patients with SAP by qPCR analysis and compared with patients with MAP and healthy controls. Concomitant to the qPCR, results of immunohistochemistry demonstrated that hfgl2 was localized on the infiltrating interstitial cells and endothelial cells of the microvasculature in the areas of inflammation in pancreatic tissues, therefore hfgl2 upregulation may be pivotal in the morbid state of SAP and lead to pathological injuries of the pancreas.

The pathway of fgl2-initiating coagulation is via ‘immunity blood coagulation’, which means fgl2 is expressed on microvascular endothelial cells, macrophages and certain other immunocytes and its expression is regulated by the mediation of a series of proinflammatory cytokines as stimuli (17,22,23). Fgl2 functions as a bridge molecule between immune and coagulation reactions. Studies have demonstrated that fgl2 was strongly expressed in endothelial cells with the induction of TNF-α (17,23), however, IFN-γ was essential for the induction of fgl2 on macrophages (23). Clark *et al* suggested that elevating fgl2 prothrombinase in trophoblasts and in the deciduas induces abortion in CBAxDBA/2 mice by stimulation of TNF-α (24–26). Based on previous studies, it may be considered that hfgl2, as an important mediator of prothrombinase activity in fibrin formation, is a key effector molecule in the pathogenesis of SAP with the induction of proinflammatory cytokines such as TNF-α as accelerators.

To determine the relevance of hfgl2 expression in PMBCs and the severity of SAP, as indicated by the Ranson scores and APACHE II within 24 h following admission, Spearman grade correlation analysis was performed. The three methods are well known for the evaluation of SAP. The results demonstrated strong correlations between hfgl2 expression in PMBCs and the assessments by the three different methods. The potential of hfgl2 measurement in PBMCs is convincing and it may be of prognostic value for predicting the severity of SAP in the early stage.

The usefulness of injection of a neutralizing antibody or gene therapy against fgl2 has been demonstrated in diseases including MHV-3 hepatitis and graft rejection, which may attenuate fibrin deposition as well as the pathological injury and prevent mice from mortality (27–30). Thus, in-depth investigation should be conducted to identify whether the inhibition of fgl2 or the application of antibodies against fgl2 are able to delay or ameliorate the course of SAP.

In conclusion, hfgl2, serving as a novel prothrombinase, and the potent function it encodes contribute to the pathogenesis of SAP. The detection of hfgl2 in PBMCs may serve as a useful marker in predicting the severity of SAP and as a promising target for therapeutic intervention.

## Figures and Tables

**Table I:** Etiology of MAP and SAP in the patients.

Etiology	No. (%) of mild cases	No. (%) of severe cases
Gallstone-associated	19 (51.4)	16 (64)
Alcohol-associated	10 (27)	5 (20)
Miscellaneous^a^	5 (13.5)	2 (8)
Unknown	3 (8.1)	2 (8)
Total	37	25

a Of the mild cases, three were diagnosed with pancreatic malignancy and two were induced post-ERCP; in the two severe cases, hyperlipidemia was detected.

MAP, mild acute pancreatitis; SAP, severe acute pancreatitis; ERCP, endoscopic retrograde cholangiopancreatography.

**Table II:** Age and gender of the three groups.

Variables	Mild (n=37)	Severe (n=25)	Healthy control (n=20)	P-value
Age (years)	59.0	62.6	58.95	0.192
Male/female ratio	17/20	11/14	9/11	0.989

**Table III:** Mean severity scores of patients with MAP and SAP.

Type of score	Mild (n=37)	Severe (n=25)	P-value
Ranson	1.54±0.61 (2)	3.64±1.19 (3)	<0.001
24-h APACHE II	6.86±1.60 (7)	10.88±2.28 (11)	<0.001

Data are expressed as the mean ± SD. Numbers in parentheses are the median values. MAP, mild acute pancreatitis; SAP, severe acute pancreatitis; APACHE, Acute Physiology and Chronic Health Evaluation.
